# Systematic investigation of functional ligands for colloidal stable upconversion nanoparticles†

**DOI:** 10.1039/c7ra13765f

**Published:** 2018-01-26

**Authors:** Hien T. T. Duong, Yinghui Chen, Sherif Abdulkader Tawfik, Shihui Wen, Maryam Parviz, Olga Shimoni, Dayong Jin

**Affiliations:** Institute for Biomedical Materials and Devices, School of Mathematical and Physical Sciences, Faculty of Science, University of Technology Sydney NSW 2007 Australia hien.duong@sydney.edu.au; ARC Research Hub for Integrated Device for End-user Analysis at Low-levels (IDEAL), Faculty of Science, University of Technology Sydney NSW 2007 Australia; School of Mathematical and Physical Sciences, Faculty of Science, University of Technology Sydney NSW 2007 Australia

## Abstract

Despite intense efforts on surface functionalization to generate hydrophilic upconversion nanoparticles (UCNPs), long-term colloidal stability in physiological buffers remains a major concern. Here we quantitatively investigate the competitive adsorption of phosphate, carboxylic acid and sulphonic acid onto the surface of UCNPs and study their binding strength to identify the best conjugation strategy. To achieve this, we designed and synthesized three di-block copolymers composed of poly(ethylene glycol) methyl ether acrylate and a polymer block bearing phosphate, carboxylic or sulphonic acid anchoring groups prepared by an advanced polymerization technique, Reversible Addition Fragmentation Chain Transfer (RAFT). Analytical tools provide the evidence that phosphate ligands completely replaced all the oleic acid capping molecules on the surface of the UCNPs compared with incomplete ligand exchange by carboxylic and sulphonic acid groups. Meanwhile, simulated quantitative adsorption energy measurements confirmed that among the three functional groups, the calculated adsorption strength for phosphate anchoring ligands is higher which is in good agreement with experimental results regarding the best colloidal stability, especially in phosphate buffer solution. This finding suggests that polymers with multiple anchoring negatively charged phosphate moieties provide excellent colloidal stability for lanthanide ion-doped luminescent nanoparticles for various potential applications.

## Abstract 2

Here we quantitatively investigate the competitive adsorption of polymers bearing phosphate, carboxylic acid and sulphonic acid anchoring groups onto the surface of UCNPs and study their binding strength to identify the best conjugation strategy.

## Graphical abstract

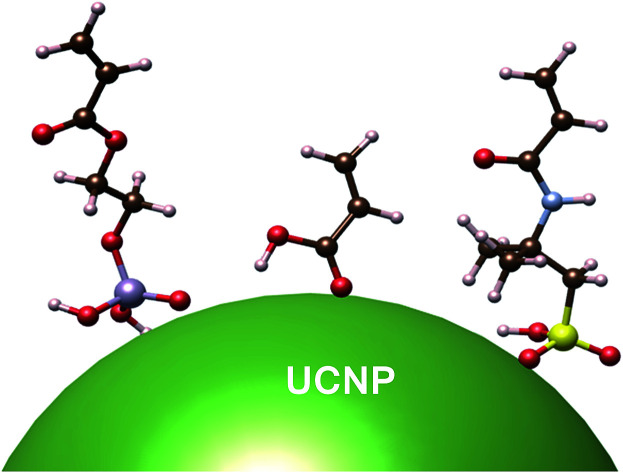

## Introduction

Rare-earth doped upconversion nanoparticles (UCNPs) are able to absorb multiple low energy photons from near-infra red (NIR) light and convert them into a high energy visible photon from the ultra-violet (UV) to the NIR region.^1–3^ Due to the unique emission properties of the photon upconversion process, UCNPs have become one of the most efficient multi-photon probes for deep-tissue light-triggered drug delivery.^4–9^ Upconversion materials consist of a crystalline host matrix doped with certain sensitizer and activator lanthanide ions determining the excitation and emission wavelength of the nanoparticles. This unique structure grants UCNPs appealing properties, including resistance to photoblinking, photobleaching, minimal photodamage and autofluorescence background in comparison with other luminescent materials (*e.g.* organic dyes, quantum dots and luminescent metal nanoclusters).^10–12^ In addition, the use of NIR light allows the deeper penetration in biological tissues that is highly favourable for many biomedical applications. NIR light penetrates tissue approximately 23 cm in depth.^13^ Over the years, UCNPs with precisely controlled size, shape and compositions have been prepared in high boiling point organic solvent.^14^ Nonetheless, for the UCNPs to become practically useful in bioapplications, it requires to overcome a critical challenge of surface functionalisation that will transform UCNPs to be stable in physiological environment and specific to recognize the target biomolecules.

The as-synthesized UCNPs are hydrophobic in nature due to their capping by long-chain hydrophobic molecules (*e.g.* oleic acid). Typically, to yield well-dispersed nanoparticles in aqueous media for bioimaging, drug delivery and other bioanalytical applications, several methods of subsequent surface modification are applied including inorganic shell, bilayer coating, ligand oxidation and ligand exchange.^15^ Despite multiple studies have been done to develop a generic approach for surface functionalization, stability of UCNPs in aqueous media, especially in phosphate buffer and physiological environment still remains as a major challenge.^16^

Among surface modification methods, polymers containing single or multiple anchoring ligands, such as hydroxyl, amine, carboxylic acid, sulphonic acid, phosphonate and phosphate groups, have been extensively studied to modify the positively charged surface of UCNPs and other inorganic nanoparticles.^15,17–19^ However, to our best knowledge, there is no report that specifically focuses on the influence of structural variation of functional ligands on the coating density, dispersity and more importantly, the extended colloidal stability of polymer functionalized UCNPs. To fill this knowledge gap, our work provides a systematic study of competitive adsorption at the nanocrystal surface between multidentate phosphate, carboxylic and sulphonic acids with oleate ligands.

Specifically, in this paper we demonstrate that a well-defined di-block copolymer synthesized by Reversible Addition Fragmentation Chain Transfer (RAFT) technique comprising of the PEG-like polymer and plurality of functional groups as potential coordination sites for the UCNP surface. RAFT polymerization technique for polymer synthesis is very simple and versatile, provides a high control over molecular weight distribution and functional groups with no tedious synthetic process.^20–23^ Multiple anchoring points are demonstrated to lead to the higher coating density on the surface of the UCNPs.^17^ PEG-like block on the polymer serves as the outer shell on the coated UCNPs that greatly assists with colloidal stability in water, serum and aqueous buffers. PEG-like polymer also provides many advantages for biological applications, including high biocompatibility, low toxicity and immunogenicity and no metabolic degradation during clearance from the body.^24^ Versatility of RAFT polymerization allows introducing a second block on the copolymer containing anchoring groups for further attachment of polymer onto the surface of UCNPs. In addition, functional end-groups of the used RAFT agent and the polymers offer an opportunity for subsequent attachments of peptide, protein, antibody, folic acid, therapeutics agents and bioactive molecules.^25–28^

Evidently, we experimentally found and verified conclusive information on the mechanism of competitive adsorption of phosphate, carboxylic and sulphonic acids with oleate ligand by analytical methods supported by simulations. In fact, the greater adsorption energy of phosphate anchoring groups is the key mechanism for the complete replacement of hydrophilic polymers which led to the best performance of the aqueous dispersity and long-term stability of phosphate containing polymer coated UCNPs.

## Experimental

### Materials and methods

All chemicals were used as received from Sigma-Aldrich, unless otherwise specified. Monomer oligo(ethylene glycol) methyl ether acrylate with average *M*_n_ of 480 g mol^−1^ (OEGA), acrylic acid (AA), monoacryloxyethyl phosphate (MAEP), and 1-acrylamido-2-methylpropane sulphonic acid (AMPS) monomers were used as received. 2,2′-Azobisisobutyronitrile (AIBN) was purified by recrystallization twice from methanol.

### Synthesis of NaYF_4_:Yb/Er upconversion nanoparticles

The synthesis of monodisperse NaYF_4_:Yb (20%), Er (2%) nanocrystals has been reported in the literature.^14^ In a typical procedure, YCl_3_ (0.78 mmol), YbCl_3_ (0.20 mmol), and ErCl_3_ (0.02 mmol) were added to a 50 mL flask containing 6 mL of oleic acid and 15 mL of 1-octadecene. The mixture was heated to 160 °C for 30 min and then cooled to room temperature. A solution of 4 mmol of NH_4_F (0.1482 g) and 2.5 mmol of NaOH (0.1 g) in 5 mL of methanol was added, and then the solution was kept at room temperature for 30 min. The mixture was then heated to 120 °C under argon for 20 min to remove methanol and water. The solution was finally heated to 300 °C under an argon atmosphere for 1.5 h and then cooled to room temperature. The nanocrystals were precipitated with 10 mL of ethanol, collected by centrifugation. The product was washed with cyclohexane, ethanol and methanol four times and the final NaYF_4_:Yb/Er nanocrystals were re-dispersed in 10 mL cyclohexane for further use.

### Synthesis of POEGA macro-RAFT agent

The RAFT agent, 2-(*n*-butyltrithiocarbonate)-propionic acid (BTPA) was prepared according to a published procedure.^29^ OEGA (5.0 g, 1.04 × 10^−2^ mol), RAFT agent (1.13 × 10^−1^ g, 4.73 × 10^−4^ mol) and AIBN (7.77 × 10^−3^ g, 4.73 × 10^−5^ mol) were dissolved in 20 mL of toluene in a round- bottom flask equipped with a magnetic stirrer bar. The flask was then sealed with a rubber septum and purged with nitrogen gas for 30 min. The reaction mixtures were then placed in a preheated oil bath at 70 °C. After 17 h, the polymerization was terminated by quenching the samples in an ice bath for 5 min. The POEGA polymer was then purified three times by precipitation with excess petroleum spirits (boiling range of 40–60 °C) followed by centrifugation (7000 rpm for 5 min) and the polymer was dried under vacuum at room temperature. The samples were stored at 4 °C until required for further chain extension to form diblock copolymers. The conversion of monomer (90%) during the course of polymerization was determined using ^1^H NMR. The molecular weight of the POEGA macro-RAFT agent was measured to be 9800 g mol^−1^ (PDI = 1.10) by DMAc GPC and *M*_n,NMR_ = 9500 g mol^−1^ by ^1^H NMR.

### Synthesis of POEGA-*b*-PAA, POEGA-*b*-PMAEP and POEGA-*b*-AMPS

POEGA with 20 repeating units (*M*_n,NMR_ = 9500 g mol^−1^, *M*_n,GPC_ = 9800 g mol^−1^) was used as a macro-RAFT agent for chain extension with three different functional monomers, acrylic acid (AA), monoacryloxyethyl phosphate (MAEP), and 2-acrylamido-2-methyl-1-propanesulphonic acid (AMPS) to yield three functional polymers POEGA_20_-*b*-PAA_5_, POEGA_20_-*b*-PMAEP_5_, POEGA_20_-*b*-PAMPS_5_ with the same length of hydrophilic block (20 repeating units) and functional block (5 repeating units). The number of repeating units of POEGA was calculated from the monomer conversion obtained from ^1^H NMR. Three reaction mixtures were prepared for the synthesis of three di-block copolymers. For the synthesis of POEGA-*b*-PAA, POEGA macro-RAFT agent (1.0 g, 1.02 × 10^−4^ mol) and AIBN (3.35 × 10^−3^ g, 2.04 × 10^−5^ mol) were dissolved in 5 mL of acetonitrile and AA (5.15 × 10^−2^ g, 7.14 × 10^−4^ mol) was added. Similar protocol was used for the synthesis of POEGA-*b*-PMAEP and POEGA-*b*-PAMPS with MAEP (1.4 × 10^−1^ g, 7.14 × 10^−4^ mol) and AMPS (1.48 × 10^−1^ g, 7.14 × 10^−4^ mol) added. *N*,*N*-Dimethylformamide (DMF) was used as the solvent for the synthesis of POEGA-*b*-PAMPS due to the insolubility of AMPS monomer in acetonitrile.

The reaction mixtures were purged with nitrogen gas for 30 min in an ice bath. The polymerization was carried out in an oil bath at 70 °C. The polymerization was terminated at the monomer conversion of around 70% by placing the samples on ice for 5 min. The di-block copolymers were purified three times by precipitation in excess of diethyl ether followed by centrifugation (7000 rpm for 5 min) and the polymer was then dried under reduced pressure at room temperature. Block copolymers with 20 repeating units of OEGA, 5 repeating units of functional monomers were obtained (as confirmed by ^1^H NMR) was used for further comparison of grafting efficiency on the surface of upconversion nanoparticles. The molecular weight was measured to be *M*_n,GPC_ = 14 700 g mol^−1^ (PDI = 1.11) and *M*_n,NMR_ = 10 200 g mol^−1^ for POEGA-*b*-PAA, *M*_n,GPC_ = 11 000 g mol^−1^ (PDI = 1.16) and *M*_n,NMR_ = 10 800 g mol^−1^ for POEGA-*b*-PMAEP, and *M*_n,GPC_ = 14 100 g mol^−1^ (PDI = 1.17) and *M*_n,NMR_ = 10 800 g mol^−1^ for POEGA-*b*-PAMPS.

### Grafting of di-block copolymers onto the surface of upconversion nanoparticles

UCNPs (20 mg) were dispersed in tetrahydrofuran (THF) (1 mL). A solution of each di-block copolymer (50 mg) in THF (2 mL) was individually added in UCNP dispersion. The resulting dispersion was sonicated for one min, followed by incubation in a shaker overnight at 40 °C. The polymer coated UCNPs were purified three times by centrifugation at 14 000 rpm for 10 min. The supernatant was removed and the nanoparticles were re-dispersed in appropriate media such as water, MES (pH 4.5) and PBS (pH 7.4) buffer for further investigation of their colloidal stability. The functionalized UCNPs were characterized using TGA, ATR-IR, TEM and DLS.

### Analytical instruments

#### NMR spectroscopy

Monomer conversion and polymer compositions were characterized by ^1^H NMR using Bruker Avance 300 (300 MHz) spectrometers in d_6_-DMSO as solvents. All chemical shifts are quoted in parts per million (ppm), referenced to residual solvent frequencies ^1^H NMR: d_6_-DMSO = 2.46 ppm. ^31^P NMR spectra of the polymer was carried out in d_6_-DMSO for POEGA-*b*-PMAEP.

Monomer OEGA conversion (*α*^OEGA^) for the first block polymer was calculated using the following equation:
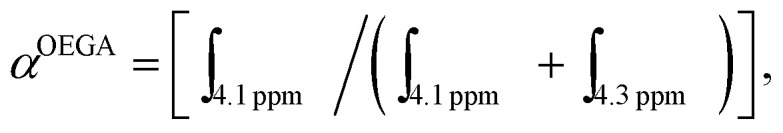
with 
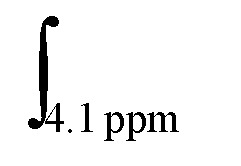
 and 
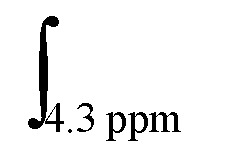
 corresponding to integral of the signal at 4.1 (polymer) and at 4.3 (monomer) ppm, respectively. The experimental molecular weight of the resultant polymer or block co-polymer can be calculated using the following equation:*M*^POEGA^_n,NMR_ = *α*^OEGA^ × ([OEGA]/[RAFT]) × *M*^OEGA^_w_ + *M*^RAFT^_w_,where *M*^OEGA^_w_ and *M*^RAFT^_w_ are the molecular weight of monomer and RAFT agent, respectively.

Monomer conversions for the second blocks (AA, MAEP and AMPS) were also calculated from ^1^H NMR spectra of the reaction mixture before and after polymerization by comparing the integral ratio of the vinyl protons of monomer and the unchanged methylene protons of the POEGA adjacent to ester bond at 4.1 ppm.

For example, AA conversion (*α*^AA^) was calculated using the following equation:
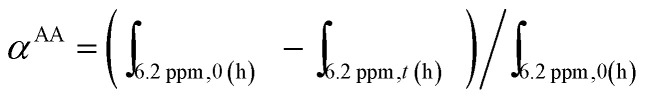
with the integrals at 4.1 ppm were set at the same value before and after polymerization.

The experimental molecular weight of the resultant polymer or block co-polymer can be calculated using the following equation:*M*^POEGA-*b*-PAA^_n,NMR_ = *α*^AA^ × ([AA]/[POEGA macro-RAFT]) × *M*^AA^_w_ + *M*^POEGA^_n,NMR_,where *M*^AA^_w_ and *M*^POEGA^_n,NMR_ are the molecular weight of monomer and macro RAFT agent, respectively.

The experimental molecular weights of the resultant POEGA-*b*-PMAEP and POEGA-*b*-PAMPS block co-polymers were calculated using the similar above approach.

#### Size exclusion chromatography (SEC)

Size exclusion chromatography (SEC) was implemented using a Shimadzu modular system comprising a DGU-12A degasser, a LC-10AT pump, a SIL-10AD automatic injector, a CTO-10A column oven, a RID-10A refractive index detector, and a SPD-10A Shimadzu UV/vis detector. A 50 × 7.8 mm guard column and four 300 × 7.8 mm linear columns (500, 103, 104, and 105 Å pore size, 5 μm particle size) were used for the analyses. *N*,*N*′-Dimethylacetamide (DMAc, HPLC grade, 0.05% w/v 2,6-dibutyl-4-methylphenol (BHT), 0.03% w/v LiBr) with a flow rate of 1 mL min^−1^ and a constant temperature of 50 °C was used as the mobile phase with an injection volume of 50 μL. The samples were filtered through 0.45 μm filters. The unit was calibrated using commercially available linear polystyrene standards (0.5–1000 kDa, Polymer Laboratories). Chromatograms were processed using Cirrus 2.0 software (Polymer Laboratories).

#### Infrared spectroscopy

ATR-FTIR spectra of the polymer grafted upconversion nanoparticles were obtained using a Nicolet 7650 system using diffuse reflectance sampling accessories. The spectrophotometer was equipped by tungsten halogen lamp and Si/Ca beam splitter. Spectra were obtained at regular time intervals in the MIR region of 4000–500 cm^−1^ at a resolution of 4 cm^−1^ (128 scans) and analysed using OPUS software.

#### Dynamic light scattering (DLS)

Dynamic light scattering studies of the upconversion nanoparticles at 0.5 mg mL^−1^ were conducted using a Malvern Instruments Zetasizer Nano ZS instrument equipped with a 4 mV He–Ne laser operating at *λ* = 633 nm, an avalanche photodiode detector with high quantum efficiency, and an ALV/LSE-5003 multiple tau digital correlator electronics system.

#### Transmission electron microscopy (TEM)

The size and dispersion of the upconversion nanoparticles was characterized using a FEI Tecnai G2 20 TEM with a beam voltage of 200 kV. Samples were prepared by placing a droplet of a 0.2 mg mL^−1^ nanoparticles solution in water on carbon-coated copper grid.

#### X-ray powder diffraction (XRD)

X-ray diffraction pattern measurements were conducted on a Bruker D8 Discover X-ray diffractometer equipped with Cu Kα radiation from 5° to 75°.

#### Thermal gravimetric analysis (TGA)

TGA was performed on a SQ600. The samples were thoroughly purified by centrifuge to remove completely the unreacted polymers. Pre-dried samples were heated from room temperature to 700 °C at a constant temperature increase of 10 °C min^−1^ using air as the furnace gas. The weight loss was calculated through the difference between the weights at room temperature and at 600 °C. The calculation of grafting density of polymers onto upconversion nanoparticles (in molecules per nm^2^) was described in ESI.†

## Results and discussion

The as-prepared upconversion nanoparticles are capped with hydrophobic oleic acid for both controlling the growth of nanocrystals during the synthesis and preventing the aggregation of the nanoparticles due to steric stabilization. For many practical applications, surface modification of nanoparticles is required for the dispersion of nanoparticles in aqueous solution. The grafting of polymer onto upconversion nanoparticles has been widely described in the literature.^15^ It is very important to point out that the choice of the right anchoring molecules is critical for the stability of polymer coated UCNPs. Theoretically, the strongly binding anchoring molecules stabilize UCNPs better than weakly binding ones. The aim of this study is to experimentally compare the adsorption competition between polymers bearing phosphate, carboxylic acid and sulphonic acid with oleate ligand to provide a better understanding of the influence of functional anchoring groups on the stability of UCNPs.

### Synthesis of POEGA-*b*-PMAEP, POEGA-*b*-PAA, POEGA-*b*-PAMPS di-block copolymer

**Scheme 1:**
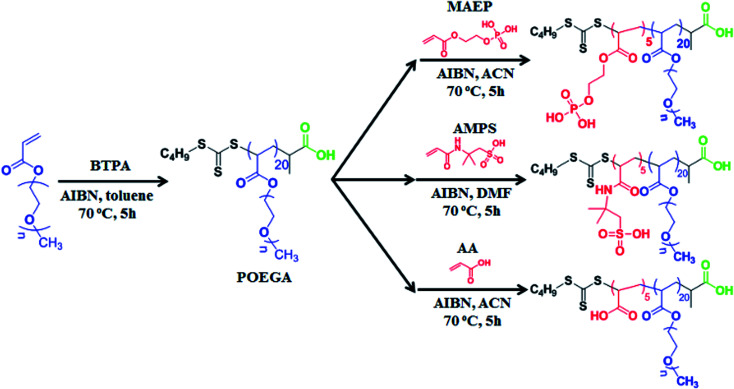
POEGA-*b*-PMAEP, POEGA-*b*-PAMPS, and POEGA-*b*-PAA di-block copolymers *via* RAFT polymerization.

We employed RAFT polymerization techniques for the synthesis of three amphiphilic di-block copolymers (POEGA_20_-*b*-PMAEP_5_, POEGA_20_-*b*-PAA_5_ and POEGA_20_-*b*-PAMPS_5_). These copolymers are composed of the same length of hydrophilic block polymer POEGA to render the biocompatibility and hydrophilicity, and a short hydrophobic block containing the five repeating units of phosphate, carboxylic acid and sulphonic acid functional groups (PMAEP, PAA and PAMPS) (Scheme 1). Poly(oligoethylene glycol) methyl ether acrylate (POEGA) macro-RAFT agent was prepared in toluene at 70 °C in the presence of BTPA RAFT agent and oligo(ethylene glycol) acrylate (OEGA) as a monomer. We determined around 90% of monomer conversion using ^1^H NMR spectroscopy. It is necessary to stop the polymerization before the full conversion to avoid the formation of significant dead polymers that will create broader molecular weight distribution. Accordingly, the molecular weight of the POEGA macro-RAFT agent was measured to be 9800 g mol^−1^ with narrow distribution (PDI = 1.10) by GPC and *M*_n,NMR_ = 9500 g mol^−1^ by ^1^H NMR (∼20 repeating units) (ESI, Fig. S1†). POEGA macro-RAFT agent has been further used for subsequent polymerization with MAEP, AA and AMPS monomers to yield POEGA-*b*-PMAEP, POEGA-*b*-PAA, and POEGA-*b*-PAMPS di-block copolymer, respectively. The synthesis of block copolymers was stopped at the conversions of these monomers to be around 70% to yield the final di-block copolymers containing additional 5 repeating units of ligand functional groups. After purification, GPC and ^1^H NMR spectra analysis confirmed the successful chains extension to a higher molecular weight with a good control of polydispersity (PDI < 1.20). We noted that the molecular weight determined by GPC for POEGA-*b*-PAA and POEGA-*b*-PAMPS are higher comparing with the values determined by ^1^H NMR. This is due to the lack of retention of these acidic polymers onto the GPC columns and also the use of linear polystyrene calibration for data analysis. Both ^1^H NMR spectra and ATR-FTIR have confirmed the presence of ester, phosphate, amide and sulphonic acid (ESI, Fig. S2–S6†). The FTIR spectra for the POEGA-*b*-PAMPS polymer shows the characteristic signals of S=O asymmetric and symmetric and S–O stretches of sulphonic groups at 1355, 1150 and 750 cm^−1^ (ESI, Fig. S6†).

### Conjugation of POEGA-*b*-PMAEP, POEGA-*b*-PAA and POEGA-*b*-PAMPS di-block copolymers onto upconversion nanoparticles

**Fig. 1:**
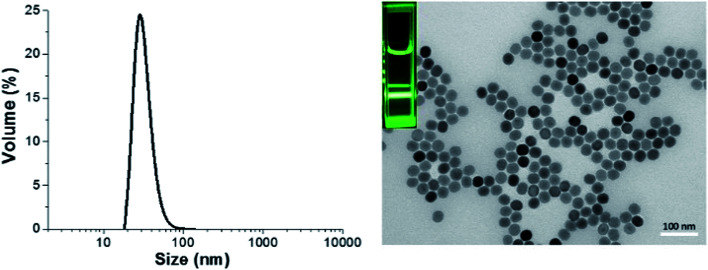
Dynamic light scattering (DLS) graph (left) and TEM image (right) depicting the size of upconversion nanoparticles in cyclohexane, luminescence imaging (inset) under 980 nm excitation.

Oleate-capped NaYF_4_:Yb/Er nanoparticles (UCNP@OA) were first synthesized following a previously reported protocol.^14,30^ TEM images of UCNP@OA showed the formation of monodisperse nanoparticles with average size of 32 nm (Fig. 1).

**Fig. 2:**
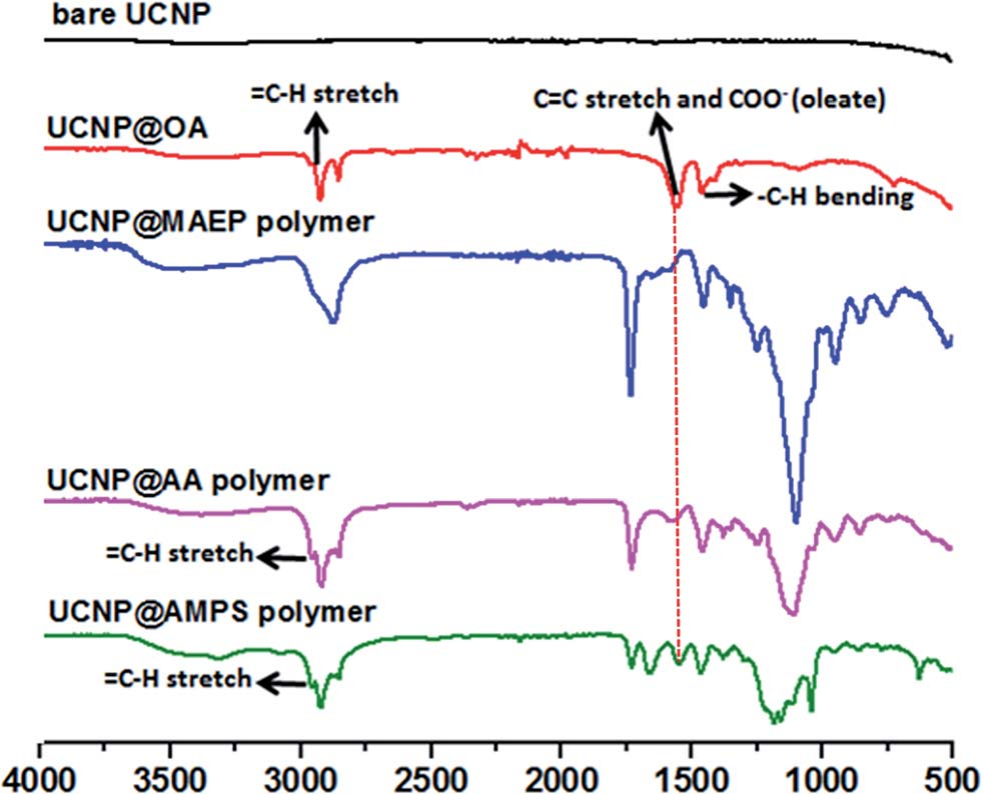
ATR-FTIR spectra of prepared bare (OA removed) UCNP, UCNP@OA and UCNP@POEGA-*b*-PMAEP polymer, UCNP@POEGA-*b*-PAMPS polymer and UCNP@POEGA-*b*-PAA polymer.

The X-Ray Diffraction (XRD) pattern of the UCNPs revealed the formation of mainly the hexagonal phase structure as bulk β-NaYF_4_ (ESI, Fig. S7†). It can be seen from the TEM image that the as-prepared OA capped UCNPs are well-dispersed in cyclohexane with no sign of aggregation and narrow size distribution was proven by DLS analysis. The prepared upconversion nanoparticles were then functionalized with di-block copolymers through ligand exchange process. The presence of copolymers on the surface of upconversion nanoparticles was confirmed using ATR-FITR and TGA techniques. ATR-FTIR is a powerful technique to prove the attachment of polymers onto the surface of UCNPs. FTIR spectra of bare UCNP, UCNP@OA, and UCNP@polymers were shown in Fig. 2. All the absorptions of UCNP@OA were clearly identified, the C–H stretching vibration bands of unsaturated –C=C–H, –CH_3_ and –CH_2_– are located at 3000, 2927 and 2852 cm^−1^ respectively, the C–H bending vibration band appears at 1443 cm^−1^ and the asymmetric and symmetric COO– stretching at 1547 and 1435 cm^−1^ of oleic acid molecules.

Interestingly, after modifying with POEGA-*b*-PMAEP polymer bearing phosphate groups, the carboxylate peak of oleic acid at 1547 cm^−1^ completely disappeared, which is confirmed by the disappearance of alkene and carboxylate signals at 3000 and 1547 cm^−1^. In contrast, di-block polymers with carboxylic acid (POEGA-*b*-PAA) and sulphonic acid (POEGA-*b*-PAMPS) functional groups only could partially replace OA as evidenced by the presence of characteristic absorptions of remaining oleate as shown in Fig. 2. This result suggests that phosphate groups on the polymer have a higher affinity to the surface of nanoparticles. It is important to emphasize that the incomplete ligand exchange greatly influences the colloidal stability in aqueous media of final polymer coated UCNPs owing to the presence of hydrophobic oleic acid molecules. Regardless, the FTIR spectra confirms the successful grafting of all three functional polymers on the surface of UCNPs by the appearance of the characteristic absorptions, such as the characteristic P=O stretches of POEGA-*b*-PMAEP (1120 cm^−1^), C=O stretches of POEGA-*b*-PAA (1730 cm^−1^) and S=O and S–O stretches of POEGA-*b*-PAMPS (1355, 1150 and 750 cm^−1^).

To quantitatively confirm the substitution amount of polymers on the surface of UCNPs, we performed thermogravimetric analysis (TGA) (ESI, Fig. S8†). The weight contribution of UCNP@OA, and UCNP@polymer nanoparticles is shown in Table 1. As the complete ligand exchange of oleic acid with POEGA-*b*-PMAEP polymer has been achieved, we are able to calculate the density of the adsorbed polymer (in molecules per nm^2^). Assuming the UCNP is spherical with the diameter of 32 nm and bulk density of NaYF_4_ is 4.2 g cm^−3^, the grafting density was calculated to be 6.73 for the OA molecules per nm^2^ for UCNP@OA and 0.29 polymer chains per nm^2^ for UCNP@POEGA-*b*-PMAEP. This is a predictable result as OA molecules 30 times lower in molecular weight than polymer and also more molecules can be adsorbed on the surface of the nanoparticles. The slightly higher weight loss for UCNP@POEGA-*b*-PAMPS and UCNP@POEGA-*b*-PAA could be attributed to the contribution of remaining oleic acid due to the incomplete ligand exchange.

**Table 1:** TGA results for UCNP@OA and UCNP@POEGA-*b*-PMAEP, UCNP@POEGA-*b*-PAA, and UCNP@POEGA-*b*-PAMPS

	Weight loss (wt%)	Grafting density (molecules per nm^2^)
UCNP@OA	12.35	6.73
UCNP@POEGA-*b*-PMAEP	14.16	0.29
UCNP@POEGA-*b*-PAA	15.32	—
UCNP@POEGA-*b*-PAMPS	17.81	—

### Colloidal stability of hybrid upconversion nanoparticles in different media

**Fig. 3:**
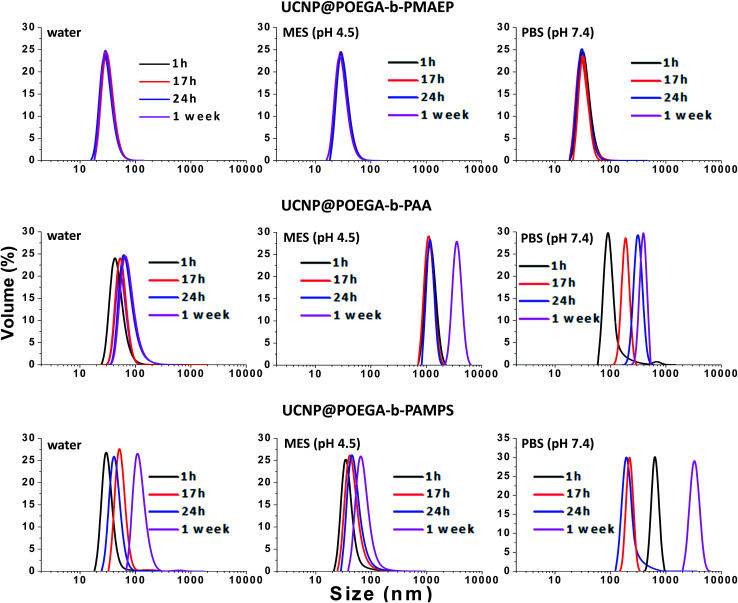
Colloidal stability of UCNP@POEGA-*b*-PMAEP polymer, UCNP@POEGA-*b*-PAMPS polymer and UCNP@POEGA-*b*-PAA polymer over one-week storage as determined by DLS measurements.

In many applications of UCNPs, one of the most important prerequisites is a good colloidal stability of nanoparticles. Indeed, the UCNP@polymers hybrid nanostructures have shown a well-dispersed behavior in ethanol, THF and other organic solvents. In what follows, we assessed the colloidal stability of the polymer coated UCNPs not only in water, but also in physiologically relevant buffers, such as MES buffer (pH 4.5) and PBS (pH 7.4) over one-week storage by measuring their hydrodynamic size using DLS. It is noted that the UCNPs can be easily dispersed in water immediately after the ligands exchange process with all three polymers and the solution is clear. After ligand exchange with POEGA-*b*-PMAEP, the UCNPs remained stable for one week without any sign of aggregation in water, PBS buffer and MES buffer (Fig. 3). The DLS plot showed the monodisperse distribution with the average size of about 38 nm after one week. Unexpectedly, the UNCPs capped with POEGA-*b*-PAA and POEGA-*b*-PAMPS, are only stable for the first few hours after transferring to water (Fig. 3). The amount of aggregation has increased over time with the shift to a higher hydrodynamic radius. After one day of storage, the UCNPs capped with POEGA-*b*-PAA and POEGA-*b*-PAMPS polymers tends to form aggregates which are visible to the naked eye. In this case, the intensity-based distribution is not fully meaningful as it only represents the large aggregates. Thus, volume-based distribution is used for the DLS data analysis to avoid the misinterpretation due to the presence of significant amount of aggregation. Both UNCPs capped with POEGA-*b*-PAA and POEGA-*b*-PAMPS have become very unstable with the severe formation of aggregation immediately after being transferred to PBS buffer (pH 7.4). UNCPs capped with POEGA-*b*-PAMPS showed better stability in MES buffer (pH 4.5) than ones capped with POEGA-*b*-PAA which could be explained by the higher acidity of sulphonic acid (p*K*_a_*ca.*−3) compared to carboxylic acid (p*K*_a_*ca.* 4.2) These DLS data demonstrated that UCNP@POEGA-*b*-PMAEP shows the best colloidal stability in aqueous media in comparison with UCNP@ POEGA-*b*-PAA and UCNP@ POEGA-*b*-PAMPS. The results correlate well with the FTIR data that show that UCNP@PAA and UCNP@PAMPS have a residual OA on the surface. The incomplete ligand exchange could be one of the reasons for the instability of UCNP@ POEGA-*b*-PAA and UCNP@ POEGA-*b*-PAMPS in aqueous media due to the hydrophobicity of OA fatty acid. Additionally, introduction of charged salt into aqueous solution also contributes to a charge screening between nanoparticles surface leading to further aggregation.

### Adsorption energy of ligand groups on UCNP surface

**Fig. 4:**
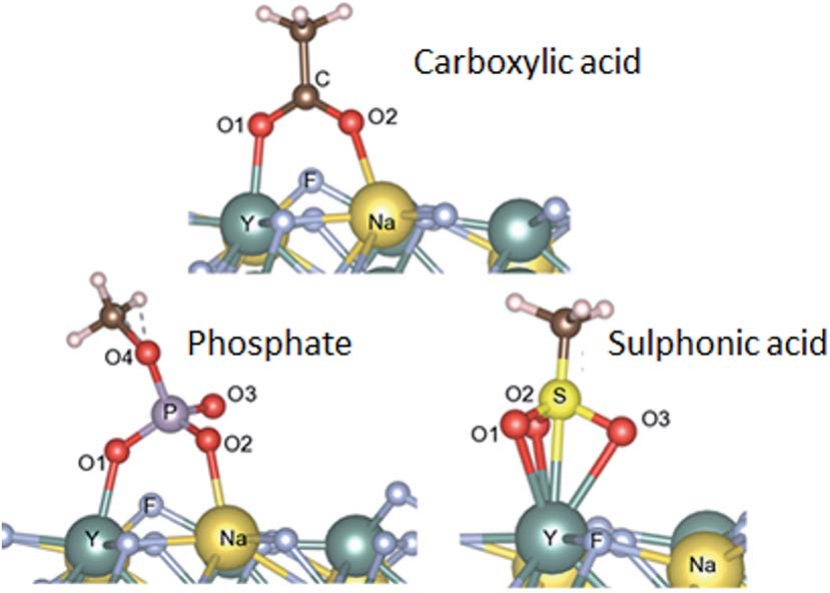
Adsorption configurations for the three structures considered in our calculations.

It is very interesting that the chemical structures of the anchoring groups strongly influence the binding of polymers onto the UCNPs and therefore their colloidal stability. To further investigate the adsorption mechanism of different functional ligands onto the surface of UCNPs, we performed density functional theory (DFT)^31^ by calculating the adsorption energies between phosphate, carboxylic, sulphonic acid groups and lanthanide ions. Hartree Fock Density Functional Theory (HF-DFT) has been used to study the adsorption of phosphonic acid on TiO_2_ surface.^32^ DFT approach is usually much simpler than HF-DFT. Specifically, we simulated the interaction between the three anchoring groups and the (001) surface of β-NaYF_4_. The specific structure for the (001) plane (ESI, Fig. S9†) was selected based on its relatively low surface energy.^14^ Details of our calculations for finding the relaxed structures are provided in the ESI.† The optimal adsorption configurations of the various groups to the surface are shown in Fig. 4. Adsorption energies and bond lengths of the deprotonated groups to the (001) surface were calculated (Table 2). Carboxylic acid and phosphate groups are bonded to the surface *via* the bonding of two O atoms to the adjacent surface Y and Na atoms, with adsorption energies of −77.9 kcal mol^−1^ and −90.4 kcal mol^−1^ respectively. The sulphonic acid group bonds by having the three O atoms bonded to one surface Y atom, with the adsorption energy of −80.0 kcal mol^−1^. It is noted that the O–Y bond lengths (Table 2) for carboxylic acid and phosphate groups are generally lower that the corresponding O–Na bond lengths, owing to the stronger bonding with the metal atoms. The adsorption of phosphate was calculated to be stronger by 12.5 kcal mol^−1^ than that of carboxylic acid group and by 10.4 kcal mol^−1^ than the sulphonic acid group. It is clear that the trend in adsorption energies agrees well with the experimental observation, where the phosphate anchor group is more competitive in binding to the surface of upconversion nanoparticles with the greatest colloidal stability.

**Table 2:** The adsorption energies of the three structures considered in the calculations and the bond lengths involving the O atoms and the Y, Na, C, P and/or S atoms. Note that the three O atoms of the SA-group are bonded to the Y atom, and therefore we list the corresponding bond lengths in the column with heading O1–Y. The S–Yt bond length (2.68 Å) is not included in this table

Ligand group	Adsorption energy (kcal mol^−1^)	Bond lengths (Å)										
		O1–Y	O2–Na	C–O1	P–O1	S–O1	C–O2	P–O2	S–O2	P–O3	S–O3	P–O4
Phosphate	−90.4	2.24	2.3	—	1.53	—	—	1.53	—	1.54	—	1.6
Carboxylic acid	−77.9	2.23	2.29	1.3	—	—	1.26	—	—	—	—	—
Sulphonic acid	−80.0	2.51, 2.50, 2.48	—	—	—	1.38	—	—	1.38	—	1.37	—

## Conclusions

The present work provides a fundamental understanding on the molecular mechanism of the competition between phosphate, carboxylic and sulphonic acid which are three widely used anchoring groups for surface modification of upconversion nanoparticles. The versatility of the RAFT polymerization technique allows us the precisely controlled composition of polymers with different ligands groups gaining an important advantage in this study and essential for conclusive comparison. Our result showed that the structural and chemical characteristics of ligand groups strongly impact the ligand exchange efficiency with OA capped UCNPs, which in turn influence the colloidal stability of the polymer capped UCNPs in aqueous media. Importantly, our experimental finding shows that phosphate ligands lead to the excellent stability of UCNPs for an extended period of time due the complete replacement of OA molecules on the surface and greater absorption energy for phosphate groups with lanthanide ions. These finding is important for many biological applications of UCNPs, especially for diagnostic and theranostics. This strategy of surface modification and functionalization would be also useful for other inorganic colloidal nanoparticles.

## Conflicts of interest

There are no conflicts to declare.

## Supplementary Material

RA-008-C7RA13765F-s001
