# Supplementary material for: Systematic investigation of functional ligands for colloidal stable upconversion nanoparticles
Source: RSC Adv. 2018 Jan 26;8(9):4842–9. doi: 10.1039/c7ra13765f (PMC9077784; doi:10.1039/c7ra13765f)
Supplement: RA-008-C7RA13765F-s001 [file RA-008-C7RA13765F-s001.pdf]

Electronic Supplementary Material (ESI) for RSC Advances.
This journal is © The Royal Society of Chemistry 2018

Supporting Information

Systematic Investigation of Functional Ligands for Colloidal Stable Upconversion Nanoparticles

Hien T.T. Duong, [a,b*] *Yinghui Chen,*[b,c] *Sherif Abdulkader Tawfik,*[d] *Shihui Wen,*[b] *Maryam Parviz,*[b,c] *Olga Shimoni* [b,c] *and Dayong Jin* [b,c]

[a] Current address: The Faculty of Pharmacy, The University of Sydney, NSW 2006, Australia

[b] Institute for Biomedical Materials and Devices, School of Mathematical and Physical Sciences, Faculty of Science, University of Technology, Sydney, NSW 2007, Australia

[c] ARC Research Hub for Integrated Device for End-user Analysis at Low-levels (IDEAL), Faculty of Science, University of Technology Sydney, NSW, 2007, Australia.

[d] School of Mathematical and Physical Sciences, Faculty of Science, University of Technology, Sydney, NSW 2007, Australia

* Authors to whom correspondence should be addressed:

Email: hien.duong@sydney.edu.au

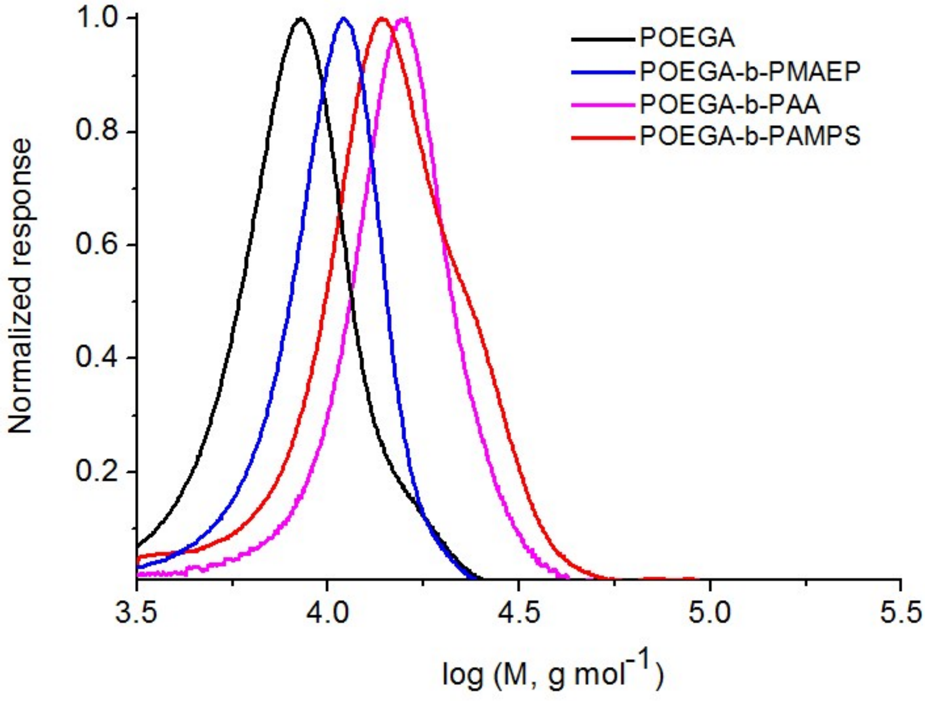

Figure S1. SEC curves of POEGA, POEGA-b-PMAEP, POEGA-b-PAMPS and POEGA-b-PAA.

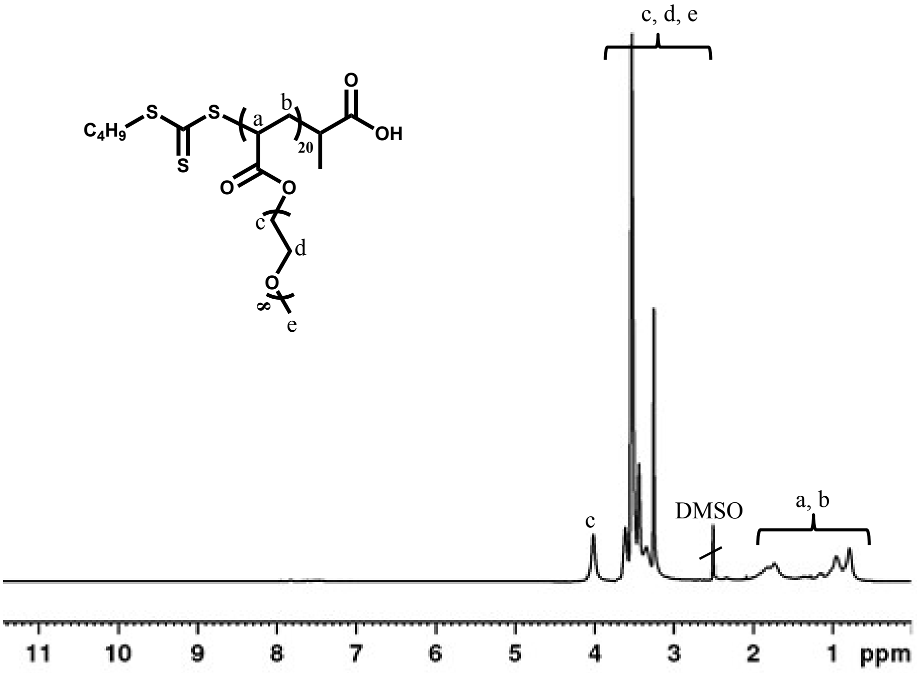

Figure S2. ^{1}H NMR spectrum of POEGA.

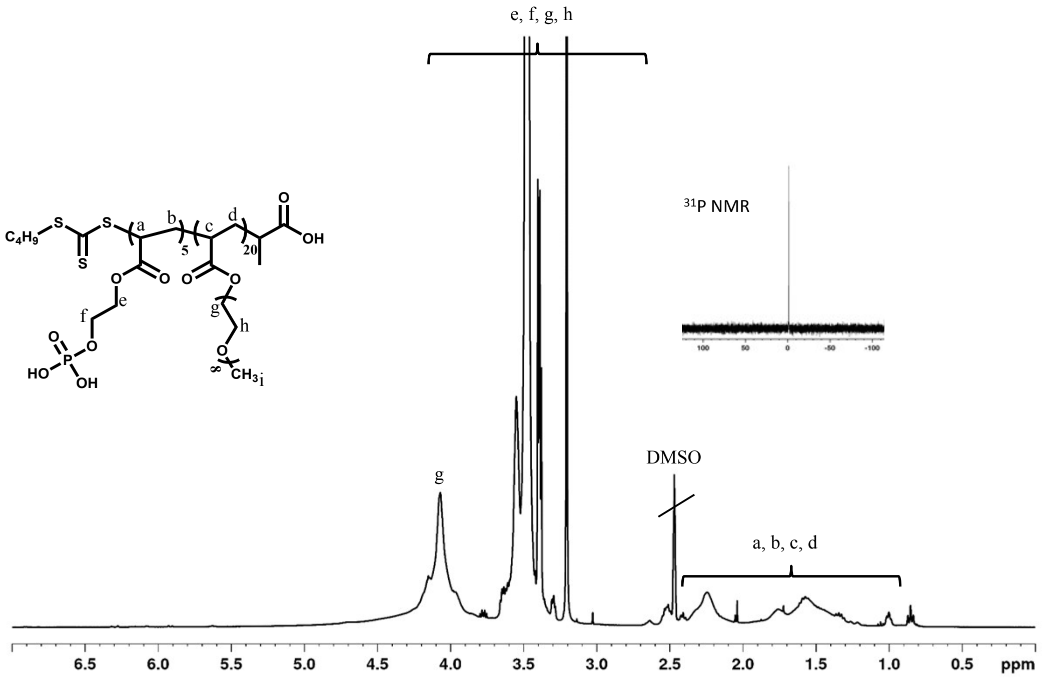

Figure S3. ^{1}H NMR spectrum of POEGA-b-PMAEP.

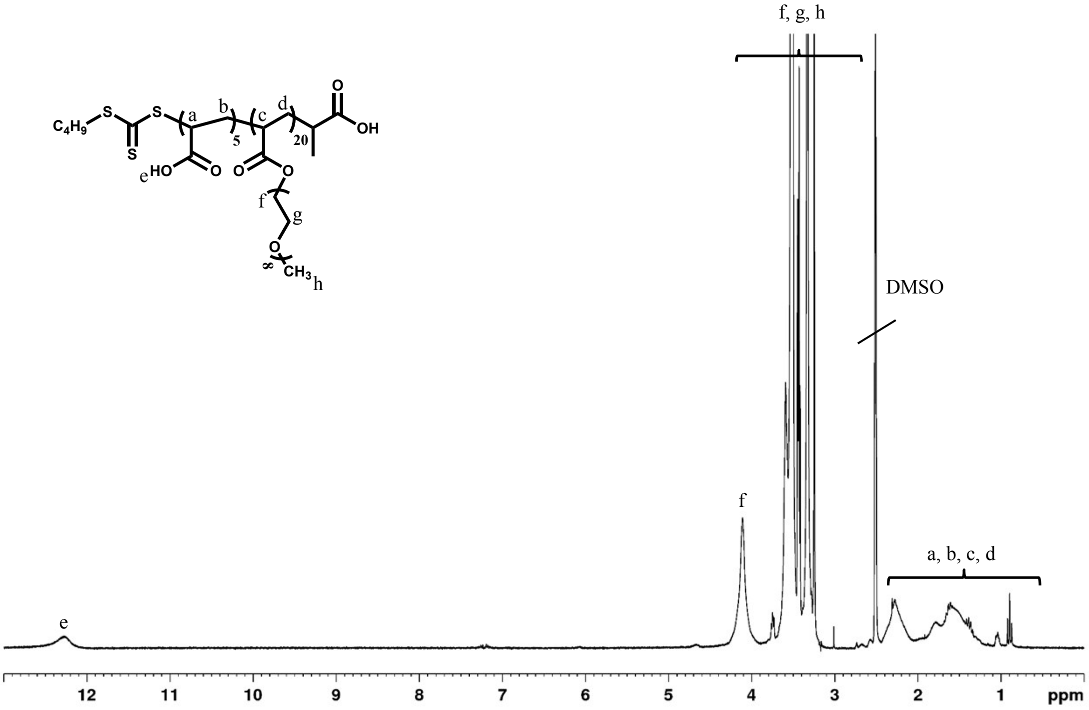

Figure S4. 1H NMR spectrum of POEGA-b-PAA.

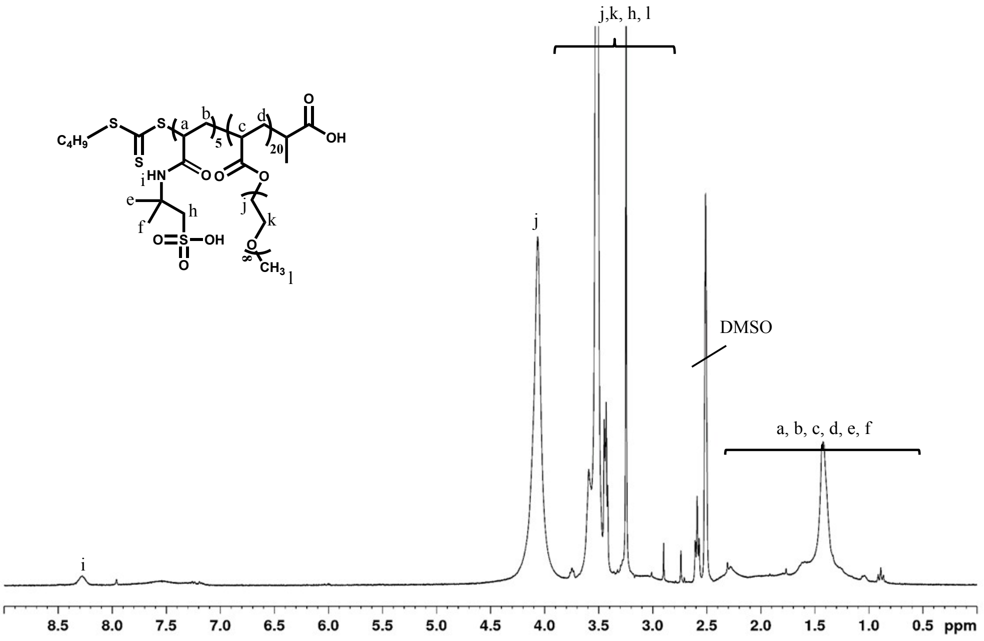

Figure S5. 1H NMR spectrum of POEGA-b-PAMPS.

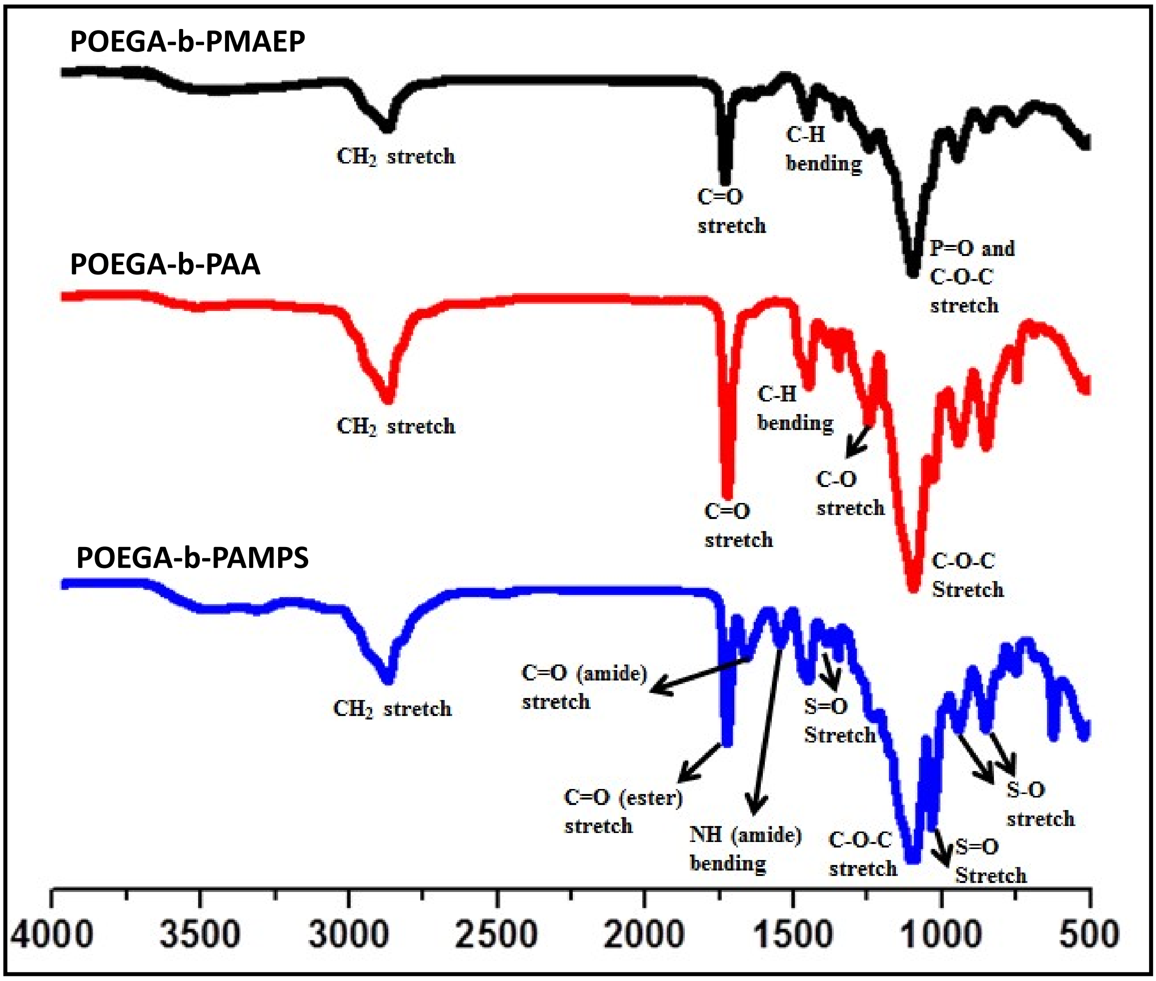

Figure S6: ATR-FTIR spectra of prepared POEGA-b-PMAEP, POEGA-b-PAMPS, and POEGA-b-PAA diblock copolymers.

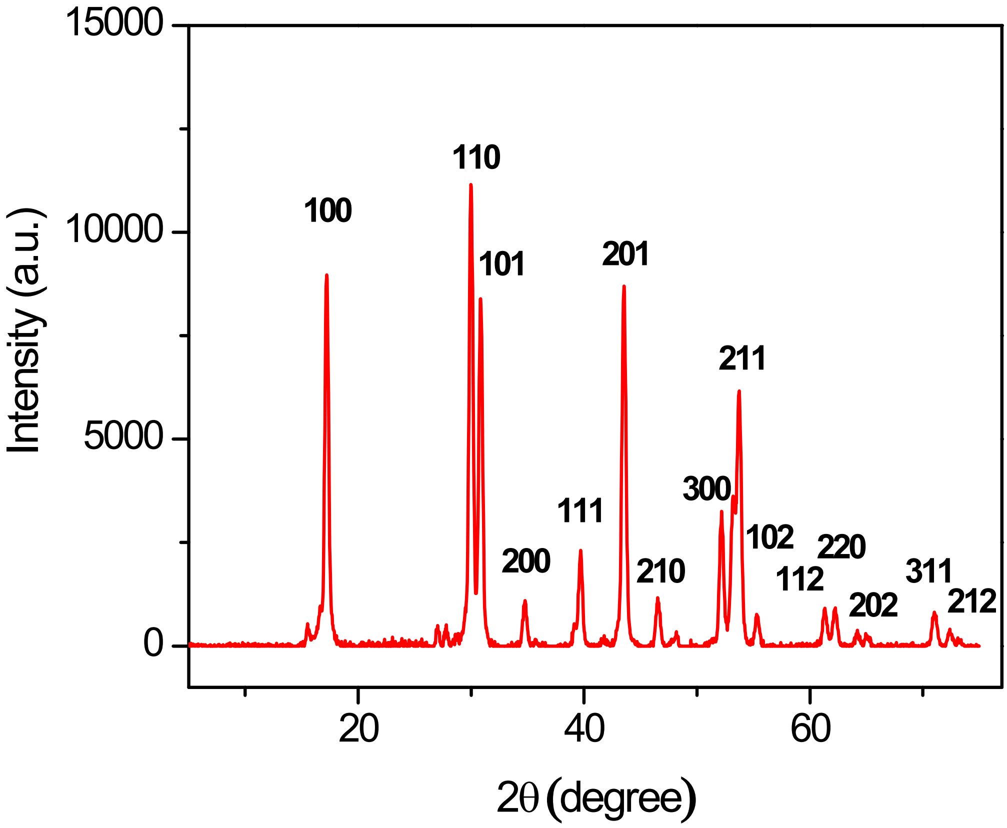

Figure S7: XRD patterns of $NaYF_4$:Yb/Er nanocrystals. X-ray diffraction pattern measurements were conducted on a Bruker D8 Discover X-ray diffractometer using Cu Kα radiation from 5° to 75°.

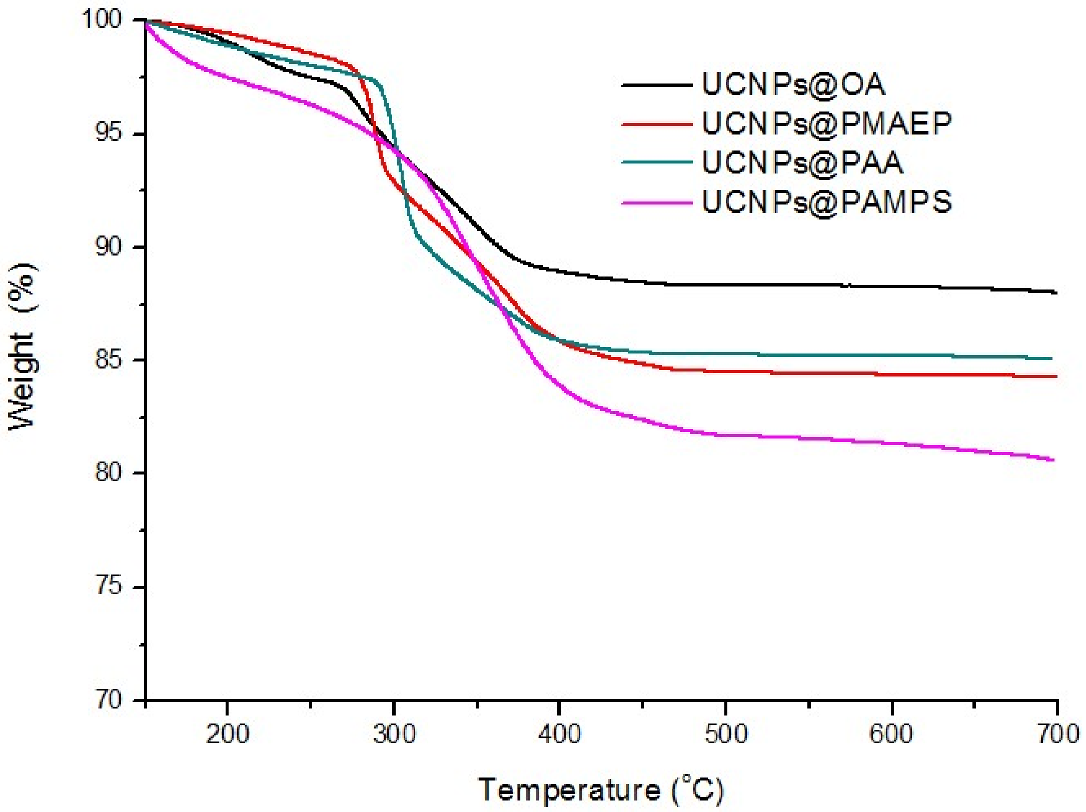

Figure S8. TGA results for UCNP@OA and UCNP@POEGA-b-PMAEP, UCNP@POEGA-b-PAMPS, and UCNP@POEGA-b-PAA.

With the assumption that the nanoparticles are spherical, the grafting density of polymers onto upconversion nanoparticles (in molecules/nm^2) was calculated from the weight loss according to the following equation:

$$Grafting\ Density = \frac{number\ of\ ligands}{surface\ area\ of\ upconversion\ nanoparticles}$$

$$Grafting\ Density = \frac{(weight^{loss}/Mn_{polymer}) \times N_A}{m_{NP} \times S_{NP}}$$

In this equation, $M_{n\ polymer}$ corresponds to the molecular weight of polymer grafted. N_A is Avogadro's number and m_{NP} is the mass of $NaYF_4$:Yb/Er upconversion nanoparticles used for the TGA analysis (e.g. mass of nanoparticles = initial mass before TGA analysis – loss of weight). Specific Surface Area (S_{NP}) of the synthesized upconversion nanoparticles was

calculated, assuming the shape to be spherical and D_{NP} is the average diameter of the nanoparticles measured by TEM to be 32 nm. The density of $NaYF_4$ nanocrystal was determined to be ρ = 4.2 g/cm3 by X-ray energy dispersive spectroscopy (XEDS) analysis.(1) Based on the equation $D_{NP\ by\ TEM} = 6/(\rho \times S_{NP})$, Specific Surface Area of the prepared upconversion nanoparticles was calculated to be 44.6 m^2/g.

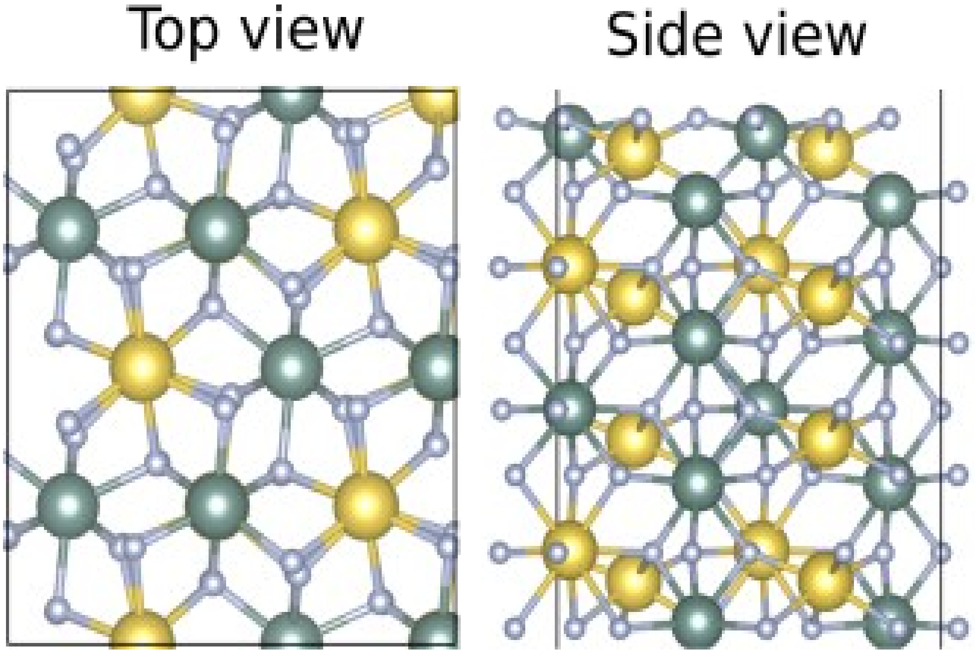

Figure S9. Top and side view of the most stable (001) surface of β-NaYF4. Blue spheres represent Y atoms, yellow spheres Na atoms and small cyan spheres F atoms.

Details of the *ab-initio* calculations

The organic acid adsorption on the β-$NaYF_4$ (001) surface was calculated in vacuum using density functional theory (DFT) within the generalized gradient approximation (GGA) of Perdew, Burke and Ernzerfof (PBE).(2) The valence electrons are separated from the core by use of projector-augmented wave pseudopotentials (PAW) (3) as implemented in the VASP package, version 5.4.1.(4) The energy cut-off for the plane wave basis set is 500 eV, and the energy tolerance is 10^{-6} to ensure the accuracy of the calculations. The lattice constants of

the β-$NaYF_4$ unit cell have been obtained as *a*=6.0 Å, *c*=3.61 Å, which is in good agreement with the experimental values of *a*=5.96 Å, *c*=3.53 Å.(5) The 144-atoms unit cell of the surface is simulated by placing it inside of a box with dimensions 10.39 Å x 12.0 Å x 30.0 Å. For the structural energy minimization, the internal coordinates are allowed to relax until all of the forces are less than 0.01 eV/Å. A vacuum region of ~ 17 Å is added between the periodic images to avoid interaction between them. The adsorption energy of group *G*, E_{ad}^{G}, is calculated by using the standard formula

$$E_{ad}^{G} = E_{Surface + G}^{G} - E_{Surface}^{G} - E_{G}$$

where $E_{Surface + G}^{G}$ is the total energy of the group *G* adsorbed on the surface, $E_{Surface}^{G}$ is the total energy of the pristine surface, and E_{G} is the total energy of the group in vacuum.

References

1. H. Liu, C. T. Xu, G. Dumlupinar, O. B. Jensen, P. E. Andersen and S. Andersson-Engels, *Nanoscale*, 2013, **5**, 10034-40.
2. J. P. Perdew, K. Burke and M. Ernzerhof, *Phys. Rev. Lett.*, 1996, **77**, 3865-8.
3. P. E. Blöchl, *Phys. Rev. B*, 1994, **50**, 17953-79.
4. G. Kresse and J. Furthmüller, *Phys. Rev. B*, 1996, **54**, 11169-86.
5. F. Wang, Y. Han, C. S. Lim, Y. Lu, J. Wang, J. Xu, H. Chen, C. Zhang, M. Hong and X. Liu, *Nature*, 2010, **463**, 1061-5.
